# Long noncoding RNA TUG1 promotes chondrosarcoma progression and M2 polarization

**DOI:** 10.1016/j.gendis.2024.101474

**Published:** 2024-11-30

**Authors:** Chao Li, Wei Wang, Binlong Zhong, Lei Zhao, Juan Li, Yihan Yu, Zhicai Zhang, Feifei Pu, Jianxiang Liu

**Affiliations:** a Department of Orthopedics, Union Hospital, Tongji Medical College, Huazhong University of Science and Technology, Wuhan, Hubei 430022, China; b Department of Orthopedics, Traditional Chinese and Western Medicine Hospital of Wuhan, Tongji Medical College, Huazhong University of Science and Technology, Wuhan, Hubei 430022, China; c Department of Orthopedics, Wuhan No.1 Hospital, Wuhan, Hubei 430022, China

**Keywords:** Chondrosarcoma, Epigenetics, EZH2, Non-coding RNA, TUG1

## Abstract

The long non-coding RNA taurine up-regulated gene 1 (TUG1) has been reported to be involved in various cancers, but its role in chondrosarcoma (CHS) remains a mystery. This research aimed to examine the function of TUG1 in CHS. We found that TUG1 expression was elevated in CHS. Functional assays demonstrated that TUG1 had a crucial role in the CHS cell progression. Mechanistically, TUG1 recruited ALYREF to maintain the stabilization of enhancer of zest homolog 2 (EZH2) mRNA and expression of H3K27me3, repressing the transcription of the tumor-suppressor gene *CPEB1*. Additionally, exosomal TUG1 enhanced the polarization of M2 tumor-associated macrophages, which increased the proliferation and metastasis of CHS. Taken together, this study revealed the oncogenic role of TUG1 in CHS and its interactions with the downstream regulatory axis, offering novel insights into the tumorigenic mechanism of CHS.

## Introduction

Chondrosarcoma (CHS) is a rare malignant tumor, with a high tendency for metastasis and resistance to both chemotherapy and radiotherapy.^1^^,^^2^ Although targeted therapies and immunotherapies have achieved positive clinical outcomes, the evidence is limited in minority populations.^3^ For the urgent need for new therapeutic approaches, the mechanism of CHS pathogenesis should be explored.

Long noncoding RNAs (lncRNAs) are a specific category of RNA molecules that consist of over 200 nucleotides.^4^ lncRNAs were widely involved in various important regulatory pathways and tumor progression.^5^, ^6^, ^7^ TUG1, one of the first identified lncRNAs, performs its oncogenic via different pathways and plays a crucial part in regulating the progression of tumor cells in some cancers such as hepatocellular carcinoma and colorectal carcinoma.^8^^,^^9^ However, the specific mechanism of TUG1 in CHS is still unclear.

The polycomb group genes (PcGs) have been recognized as transcriptional repressors, which consist of two PcG protein core complexes: polycomb repressive complex 1 (PRC1) and polycomb repressive complex 2 (PRC2).^10^^,^^11^ EZH2, a catalytic subunit of PRC2, is responsible for catalyzing the trimethylation of lysine residue 27 on histone 3, which in turn regulates gene transcription.^12^ Niu et al found that TUG1 played a role in the proliferation and resistance to chemotherapy of small cell lung cancer by modulating LIMK2b through EZH2.^13^ In acute myeloid leukemia, TUG1 conferred adriamycin resistance to acute myeloid leukemia cells by epigenetically silencing miR-34a and recruiting EZH2.^14^ However, the mechanism of TUG1 and EZH2 in CHS remains unknown.

Our research revealed that TUG1 expression was elevated in CHS, which repressed the expression of CPEB1 by EZH2. The exosome-derived TUG1 could induce tumor-associated macrophages into M2-like polarization, thus accelerating the progression of CHS.

## Materials and methods

### Cell culture and treatment

The C-28/I2 chondrocyte cell line and HCS-2/8 CHS cell line were acquired from the CBCAS (Shanghai, China) and cultured in DMEM containing 10% fetal bovine serum. OUMS-27 cells were acquired from JCRBCB (Tokyo, Japan) and cultured in DMEM containing 10% fetal bovine serum. SW1353 and JJ012 cells were sourced from ATCC (Manassas, VA, USA). SW1353 cells were grown in an L-15 medium, while JJ012 cells were kept in DMEM. All the cells were cultured in a humidified incubator with 5% CO_2_.

### RNA isolation and RT-qPCR

Total RNA was isolated from CHS cells with Trizol reagent (Invitrogen, USA) according to the manufacturer’s guidelines. The PrimeScript RT reagent kit (Takara, USA) was utilized to convert RNA into cDNA through reverse transcription. The SYBR Green Kit from Thermo Fisher in the USA was utilized to conduct quantitative real-time PCR (RT-qPCR) analysis. Data was standardized based on the levels of GAPDH or U6 utilizing the 2^−ΔΔCT^ technique. The primer sequences utilized in this research are detailed in Table S1.

### Plasmid construction and transfection

GenePharma (Shanghai, China) synthesized short hairpin RNA (shRNA) directed against TUG1, EZH2, DNMT3B, and ALYREF, and a control shRNA. The Lipofectamine™ 3000 Transfection Reagent (Invitrogen) was utilized for cell transfection following the guidelines provided by the manufacturer. Additional tests were conducted 48 h post transfection.

Lentivirus was utilized to conduct gene overexpression. PCR was used to amplify the TUG1 and EZH2 genes from the cDNA of SW1353 cells. Initially, TUG1 and EZH2 were introduced into 293T cells via Lipofectamine 3000 (Invitrogen) to generate a lentivirus that overexpressed TUG1 or EZH2, in accordance with the manufacturer’s guidelines. The primers used are listed in Table S1.

### Nuclear-cytoplasm separation and fluorescence *in situ* hybridization (FISH) assay

Cells were collected and washed thrice with phosphate buffer saline. The nuclear-cytoplasm separation test was conducted with the PARIS kit from Life Technologies in the USA, following the provided guidelines. RT-qPCR was utilized to quantify the levels of TUG1, U6, and GAPDH expression. Custom FISH probes targeting TUG1 and EZH2 were created by Servicebio Technology in Wuhan, China. RNA FISH tests were conducted with the Ribo FISH kit (RiboBio Inc., China) to examine where TUG1 was located within cells and if TUG1 and EZH2 were colocalized in CHS cells. All images were analyzed on a fluorescence microscope.

### RNA immunoprecipitation (RIP) and pull-down assays

The RIP assays were performed with an RIP kit from Millipore. CHS cell lysates (1 × 10^7^) were acquired with complete RIP lysis buffer and then subjected to immunoprecipitation using RIP buffer with anti-EZH2 antibody or anti-IgG. Subsequently, magnetic beads were mixed in CHS lysates. The extracted RNA was analyzed by RT-qPCR.

TUG1-binding proteins were examined by RNA pull-down experiments. The obtained protein was separated from the RNA-protein compound and measured using silver staining.

### Chromatin immunoprecipitation assay

SW1353 and JJ012 cell lines (2 × 10^6^ cells per experiment) were treated with 1% formaldehyde at room temperature for 10 min and then neutralized with 125 mM glycine for 5 min. The interconnected chromatin was fragmented using sonication with a Bioruptor plus to produce DNA fragments with an average length of 200–500 bp. Antibodies against normal mouse or rabbit IgG were used to immunoprecipitated chromatin fragments. The precipitated DNA was cleansed and concentrated with the ChIP DNA Clean & Concentrator Kits (Zymo Research).

### Immunofluorescence assay

The deparaffinized and rehydrated tissues or cells were placed on glass slides and then treated with 4% formaldehyde for 30 min. After that, they were blocked with 5% normal goat serum for 1 h, permeabilized with 0.5% Triton X-100 for 15 min, and finally incubated to primary antibodies at 4 °C overnight, followed by incubation with secondary antibodies for 30 min. Cell nuclei were labeled using DAPI (Sigma–Aldrich) at ambient temperature for 30 min. Immunofluorescence pictures were captured with a ZEISS Axio Scope A1 upright microscope.

### Immunohistochemistry staining

Xylene and ethanol were used to deparaffinize and rehydrate all tumor sections that were formalin-fixed and paraffin-embedded. The endogenous peroxidase was inactivated by 3% H_2_O_2_. After blocking with goat serum (C-0005, Bioss) for 30 min, the sections were incubated with antibodies targeting Ki67, EZH2, CPEB1, CD206, and CD163 at 4 °C overnight, followed by incubation with the secondary antibody IgG and Maye’s hematoxylin at room temperature for 30 min. Immunostaining images were obtained under an Olympus BX51 microscope (Olympus, Tokyo, Japan). Results were analyzed by two pathologists independently.

### Flow cytometry

For the identification of macrophage surface markers, cells were suspended in cold phosphate buffer saline and then exposed to anti-human CD206 or anti-human CD163 at 4 °C for 30 min in the dark. Subsequently, cells with labels were analyzed using a FACScan flow cytometer (BD Biosciences, San Jose, CA, USA).

### Western blotting

Cells were washed with phosphate buffer saline after 24 h–48 h. Next, 80 μL of loading buffer with protease and phosphatase inhibitors was added. Cell lysates were scraped down and sonicated using Bioruptor Plus 30 S with low power thrice. After boiling the extraction at 100 °C for 10 min, the protein was separated through 10% SDS-PAGE; subsequently, the protein was transferred to PVDF membranes. The loaded membranes were blocked with 5% skimmed milk and exposed to primary antibodies against EZH2 (GTX82503, GeneTex), ALYREF (GTX113917, GeneTex), E-cadherin (GTX50757, GeneTex), and N-cadherin (GTX127345, GeneTex) at 4 °C overnight. Protein bands were exposed to secondary antibodies conjugated with horseradish peroxidase (MultiSciences, GAM0072, and GAR007) and detected using enhanced chemiluminescence (Merck Millipore, Billerica, USA).

### Invasion and migration assays

CHS cells were placed in the top inserts with serum-free medium, while 600 μL of medium containing 10% fetal bovine serum was introduced into the nether chamber (3422#, Corning, USA). After 24-h incubation in a moist environment at 37 °C and 5% CO_2_, the cells in the top inserts were removed using cotton swabs, while the cells in the lower inserts were treated with 0.25% crystal violet stain for 30 min, followed by imaging.

### Exosome isolation and experiments

CHS cells were cultured in a medium supplemented with 10% exosome-free fetal bovine serum for 48 h, and then cell culture supernatant was collected to remove large vesicles. A total exosome isolation reagent (Invitrogen, USA) was applied to isolate exosomes in CHS cells according to the manufacturer’s instructions. Transmission electron microscopy (FEI Tecnai G20 TWIN) was applied to observe the morphology of exosomes. Exosome particle size was detected using nanoparticle tracking analysis via the NANOSIGHT NS300 system (Malvern, UK).

### Cell counting kit-8 assay (CCK8)

CHS cells were placed in suspension and incubated in a 96-well plate with 100 μL medium for 24, 48, and 72 h. Subsequently, the medium was supplemented with the CCK8 reagent as per the instructions provided by the manufacturer. Cells were incubated at 37 °C for 4 h. Optical density at 450 nm was measured by a microplate reader.

### *In vivo* assays

The Ethics Committee reviewed and approved this study. Male BALB/C nude mice were used to construct subcutaneous CHS and CHS metastasis models. The mice were divided into two sets, each set containing seven mice, in a random manner. To ensure the precision and consistency of results, each group was assigned five mice, with an additional two mice serving as backups. When performing the tumor samples *in vitro*, the investigators were unaware of the group assignment.

One set of mice received a subcutaneous injection of 5 × 10^6^ SW1353 cells transduced with shNC, while another set received an injection of 5 × 10^6^ SW1353 cells transduced with shTUG1. The size of the tumor was assessed every four days by measuring with a caliper, and the area was calculated (length × width)^2^/2. Following a period of 28 days, mice were euthanized by inhaling carbon dioxide, after which their tumors were surgically excised for volume measurement. Part of the tumors was immediately fixed in polyformaldehyde for immunohistochemistry analysis or freezing for RNA extraction.

To study the metastasis of CHS to the lung or liver, 5 × 10^5^ SW1353 cells with or without TUG1 knockdown were injected into the tail veins of mice. Mice were euthanized by inhalation of CO_2_ after 28 days of injection. Counting was done on the number of metastasis nodules. Then the obtained lung and liver tissues from mice were used for hematoxylin and eosin staining to examine the metastasis.

### Statistical analysis

Data analysis was performed on Excel and GraphPad Prism 9. Every test was conducted with a minimum of three separate duplicates. The mean ± standard deviation was used to display quantitative findings. The student’s *t*-test or one-way analysis of variance (ANOVA) was used to determine the statistical significance of two or more groups. The statistical significance level was set as *p* < 0.05.

## Results

### TUG1 expression is up-regulated in CHS

**Figure 1:**
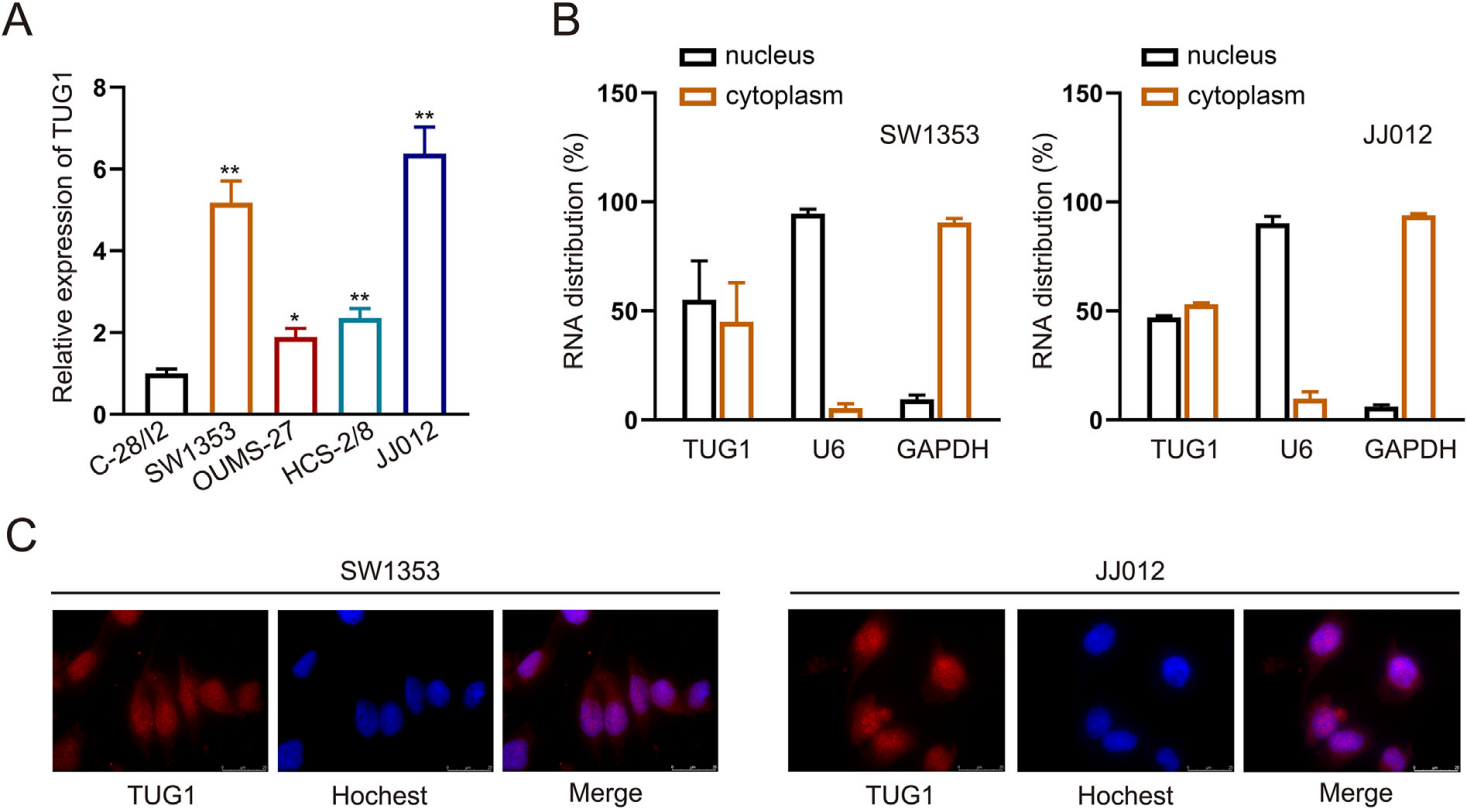
lncRNA TUG1 was overexpressed in chondrosarcoma. **(A)** Quantitative real-time PCR was performed to measure TUG1 expression in chondrocyte cell and chondrosarcoma cell lines. **(B)** Detection of subcellular location of lncRNA–TUG1 in both SW1353 and JJ012 cell lines. **(C)** The fluorescence *in situ* hybridization experiment was conducted to measure the expression of TUG1. The data is represented as mean ± standard deviation; *n* = 3; ∗*p* < 0.05, ∗∗*p* < 0.01.

To investigate the function of TUG1 in CHS, we initially examined the levels of TUG1 in chondrocytes and various CHS cell lines through RT-qPCR analysis. As shown in Figure 1A, compared with normal chondrocytes, CHS cells had significantly higher expression of TUG1. SW1353 and JJ012 cells had the highest expression; thus, they were used for subsequent experiments.

The precise way in which lncRNAs exert regulatory influence is frequently dependent on where they are located within the cell. Thus, we investigated the cellular localization of TUG1. Subcellular fractionation and FISH assays showed that TUG1 was located in both the cytoplasm and nucleus (Fig. 1B, C).

### lncRNA TUG1 exerts oncogenic roles in CHS progression

**Figure 2:**
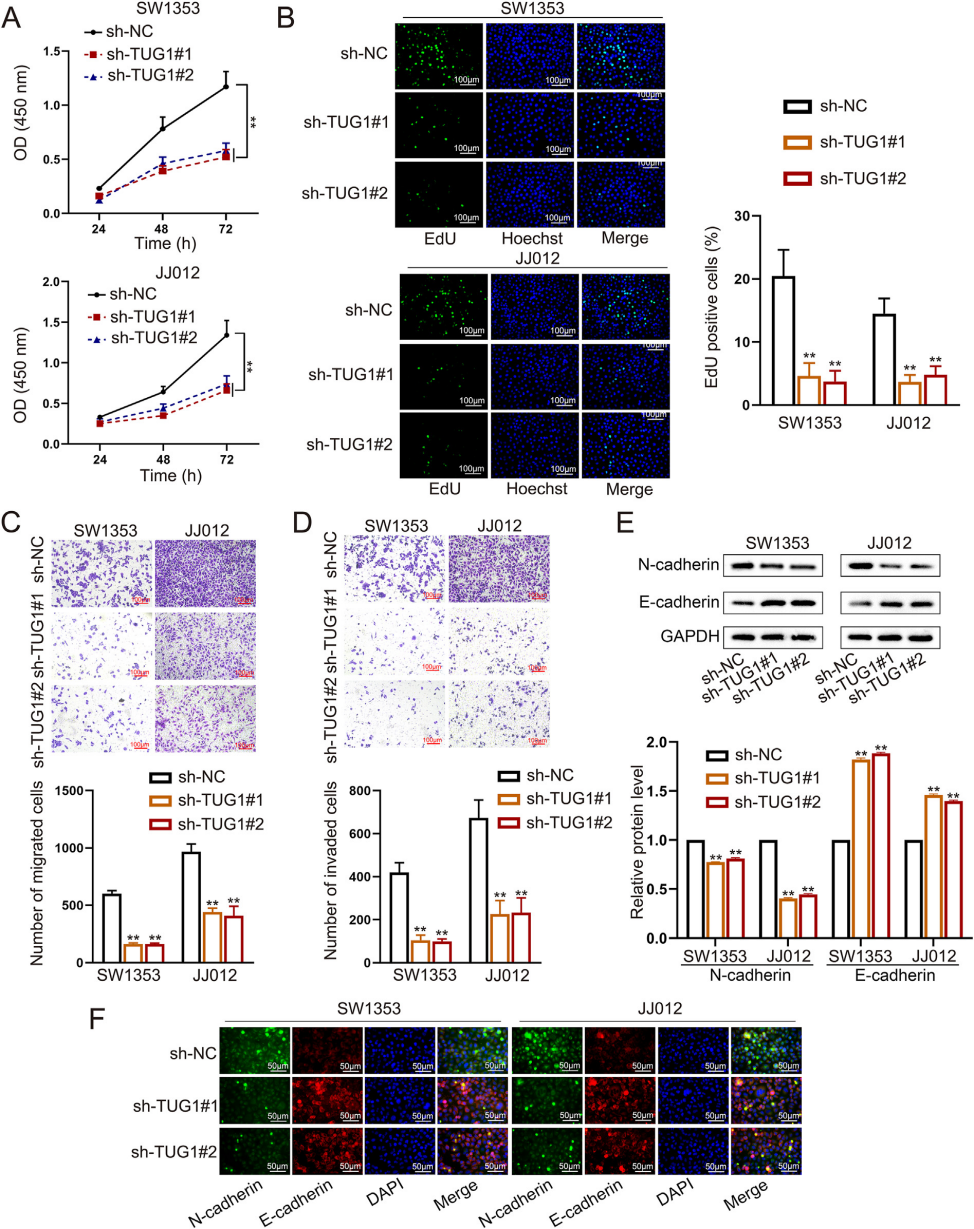
TUG1 knockdown inhibits the proliferation, migration, and invasion of chondrosarcoma (CHS) cells. **(A)** CCK8 assays showed the proliferation ability of CHS cells with or without TUG1 knockdown. **(B)** EDU experiments showed the proliferation ability of CHS cells. **(C, D)** Transwell experiments showed the migration and invasion abilities of CHS cells after transfection of shNC or shTUG1. **(E, F)** Western blotting and immunofluorescence assays were applied to measure the expression of N-cadherin and E-cadherin in SW1353 and JJ012 cells with or without TUG1 knockdown. GAPDH was used as a control group. The data is represented as mean ± standard deviation; *n* = 3; ∗*p* < 0.05, ∗∗*p* < 0.01.

To confirm the function of TUG1 in CHS, we efficiently knocked down *TUG1* in SW1353 and JJ012 cell lines (Fig. S1A). We also designed one shNC as the negative control and then transduced shTUG1 and shNC into SW1353 and JJ012, respectively. Next, we assessed the cell proliferation, migration, and invasion with or without TUG1. TUG1 knockdown led to a notable decrease in CHS proliferation as shown by the CCK8 assay (Fig. 2A). Both SW1353 and JJ012 cell lines transfected with TUG1 shRNA had fewer positive cells than the shNC group in the EdU assay (Fig. 2B). The CCK8 and EdU assays confirmed that TUG1 was required for the proliferation of CHS.

The Transwell experiments were conducted to assess the impact of TUG1 on the migration and invasion of CHS cells, revealing that TUG1 knockdown reduced their migration and invasion (Fig. 2C, D). Subsequently, the levels of E-cadherin and N-cadherin were assessed in CHS cells with or without TUG1 knockdown. The Western blot analysis revealed that silencing TUG1 led to the up-regulation of E-cadherin and down-regulation of N-cadherin. Immunofluorescence analysis showed consistent results (Fig. 2E, F).

### TUG1 interacts with EZH2 in CHS

**Figure 3:**
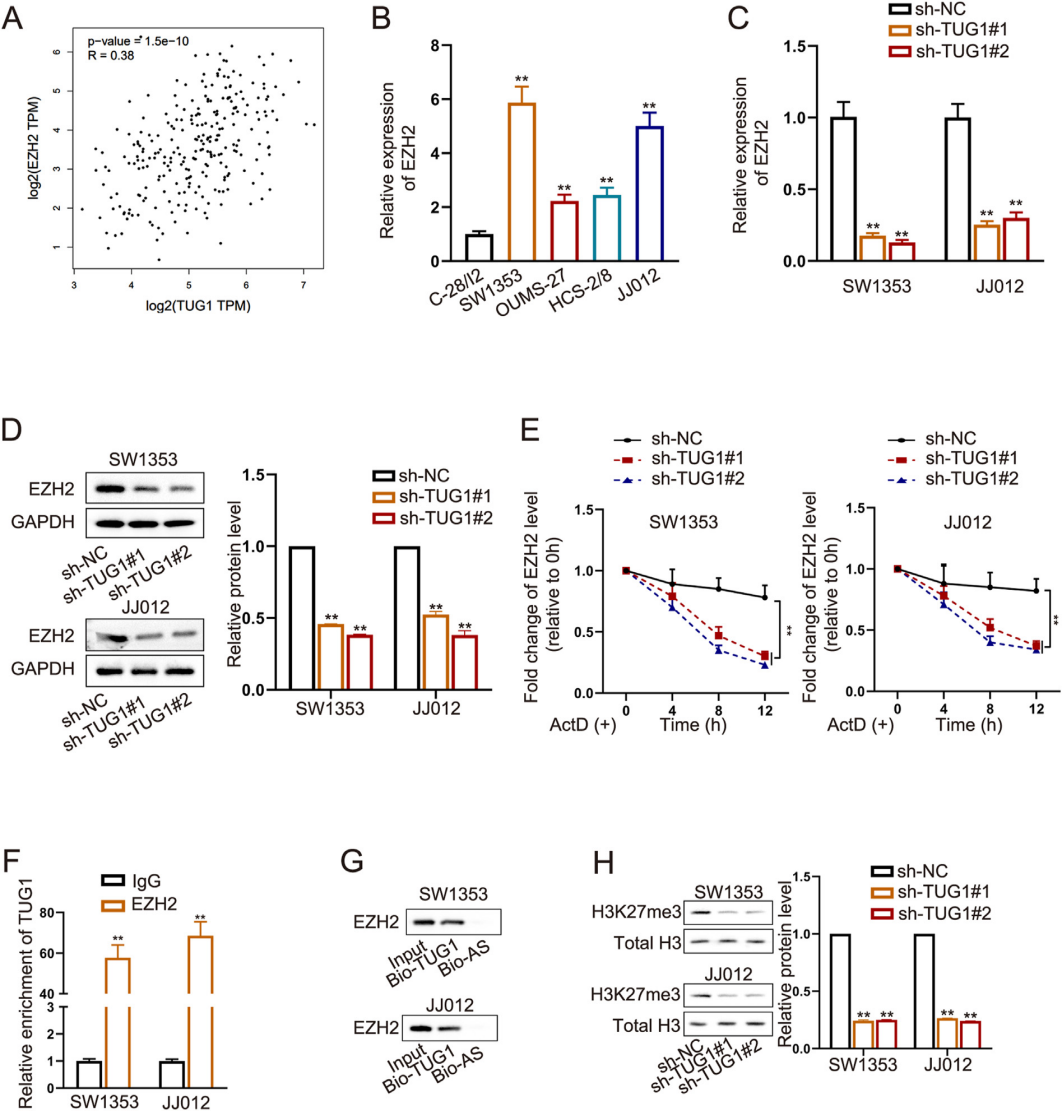
TUG1 interacts with EZH2 in chondrosarcoma (CHS). **(A)** The GEPIA database showed a positive correlation between TUG1 and EZH2 expression in SARC tissues. **(B)** Quantitative real-time PCR data showed the levels of EZH2 mRNA in normal chondrocyte cells and CHS cells. **(C, D)** Quantitative real-time PCR analysis (C) and Western blot (D) results of EZH2 levels under TUG1 knockdown versus control in CHS cells. **(E)** RNA decay assays in TUG1 knockdown CHS cells treated with actinomycin D. **(F)** RNA immunoprecipitation analysis demonstrated the interaction of EZH2 with TUG1. **(G)** RNA pull-down assay confirmed that TUG1 could pull down EZH2 (Bio-TUG1: biotinylated TUG1; Bio-AS: biotinylated antisense RNA). **(H)** Protein expression in TUG1 knockdown CHS cells was analyzed by western blotting. The data is represented as mean ± standard deviation; *n* = 3; ∗*p* < 0.05, ∗∗*p* < 0.01.

Several research studies have shown that different lncRNAs can stimulate EZH2 to trigger the expression of specific genes, thereby modulating their transcription.^15^^,^^16^ As a result, we hypothesized that TUG1 may bind to EZH2 to enhance the advancement of CHS. Initially, we utilized the GEPIA database to examine the levels of TUG1 and EZH2 in sarcoma, revealing a direct relationship between EZH2 and TUG1 expression (Fig. 3A). Next, we conducted RT-qPCR to assess the EZH2 expression in four CHS cells; in comparison to chondrocytes, there was a notable increase in EZH2 levels in all CHS cell lines (Fig. 3B). Among the four cell lines, SW1353 and JJ012 cells had the highest expression of EZH2, similar to TUG1 expression.

Subsequently, RT-qPCR and Western blot experiments were performed to assess the expression of EZH2 in cell lines with TUG1 knockdown. CHS cells with TUG1 knockdown exhibited decreased levels of EZH2 expression at both the mRNA and protein levels (Fig. 3C, D). RIP (Fig. 3F) and RNA pull-down (Fig. 3G) assays verified the connection between TUG1 and EZH2. The half-life of EZH2 was significantly shorter in the TUG1 knockdown set than in the shNC set (Fig. 3E). Considering the transcriptional regulation role of EZH2, we conducted western blotting analysis using anti-H3K27me3 antibodies to measure the transcriptional role of EZH2 in TUG1 knockdown SW1353 and JJ012 cells. Tri-methylation of histones was reduced upon TUG1 knockdown (Fig. 3H). Collectively, these data demonstrated that TUG1 interacted with EZH2 to regulate its stability.

### TUG1 stabilizes m5C-methylated EZH2 by recruiting ALYREF

**Figure 4:**
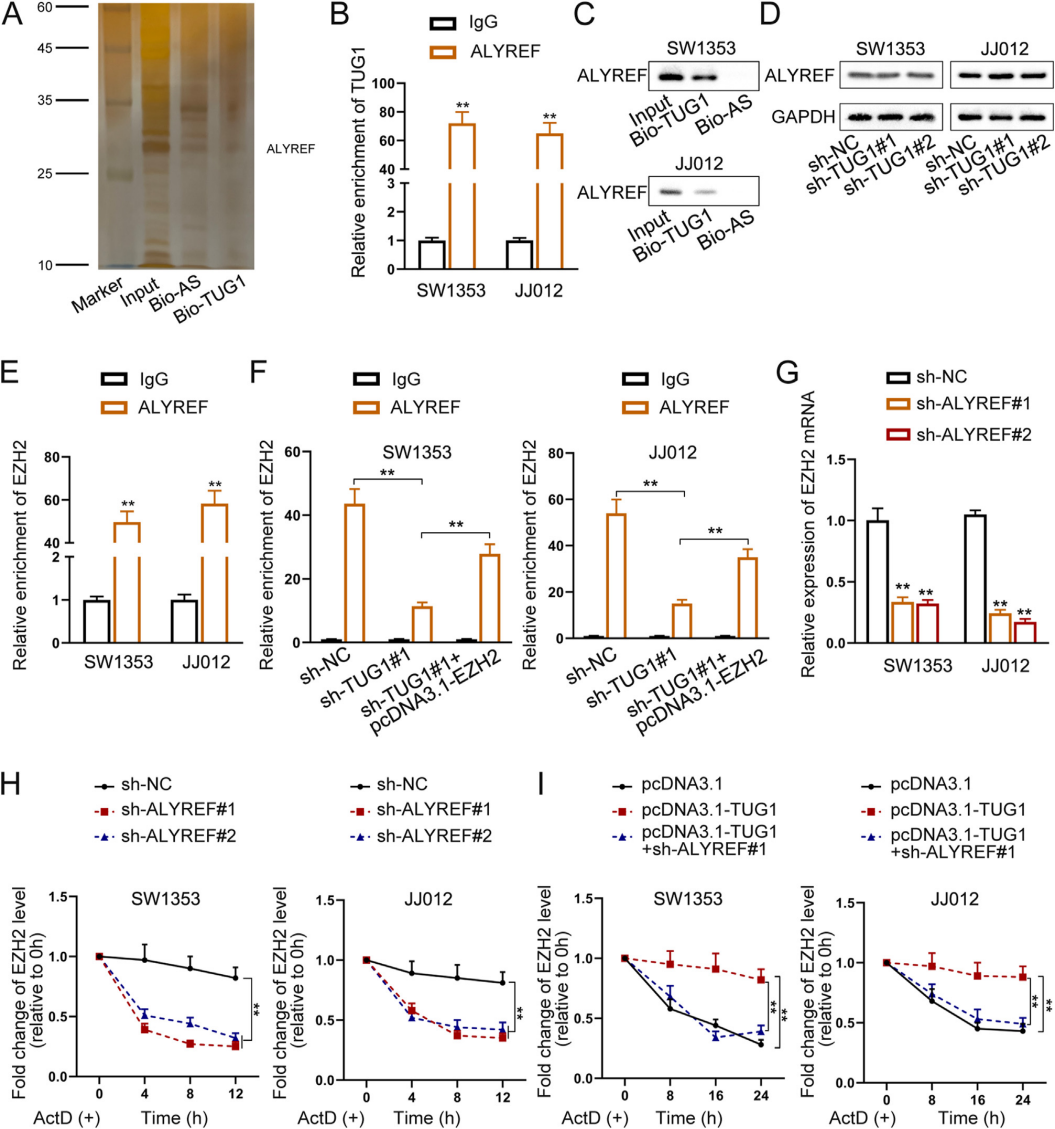
TUG1 increases EZH2 mRNA stability via recruiting ALYREF. **(A)** Mass spectrometry after silver staining experiments confirmed the binding of TUG1 to ALYREF (Bio-TUG1: biotinylated TUG1; Bio-AS: biotinylated antisense RNA). **(B)** RNA immunoprecipitation analysis showed the co-immunoprecipitation of ALYREF and TUG1. **(C)** RNA pull-down experiment demonstrated that ALYREF could be pulled down by TUG1 (Bio-TUG1: biotinylated TUG1; Bio-AS: biotinylated antisense RNA). **(D)** Western blot results of the expression of ALYREF in chondrosarcoma (CHS) cells with or without TUG1 knockdown. **(E)** RNA immunoprecipitation analysis demonstrated the co-immunoprecipitation of ALYREF and EZH2 mRNA. **(F)** RNA immunoprecipitation analysis showed the enrichment levels of ALYREF and EZH2 mRNA in SW1353 and JJ012 cells under indicated transfection. **(G)** Quantitative real-time PCR results of EZH2 levels under ALYREF knockdown versus control in CHS cells. **(H)** Half-life of EZH2 in ALYREF-depleted CHS cells treated with actinomycin D. **(I)** Half-life of EZH2 in TUG1 overexpression CHS cells (with or without ALYREF knockdown) treated with actinomycin D. The data is represented as mean ± standard deviation; *n* = 3; ∗*p* < 0.05; ∗∗*p* < 0.01.

To further reveal the mechanism by which TUG1 stabilizes EZH2, we performed RNA pull-down assays followed by mass spectrometry and silver staining. TUG1 was bound to the m5C reader ALYREF, a well-known RNA-binding protein (Fig. 4A), which has oncogenic roles in multiple cancers.^17^^,^^18^

The RIP assay using control IgG and anti-ALYREF antibodies verified the interaction between ALYREF and TUG1 in CHS cells (Fig. 4B). The interaction between TUG1 and ALYREF was also validated through an RNA pull-down assay using biotinylated TUG1 or biotinylated antisense RNA (Fig. 4C). Western blotting analysis showed that the knockdown of TUG1 did not affect the protein level of ALYREF (Fig. 4D).

To examine the functional relationship between ALYREF and EZH2, we conducted RIP assays to validate the binding of ALYREF to EZH2 mRNA (Fig. 4E). After TUG1 knockdown, the enrichment of EZH2 mRNA was significantly reduced, whereas following EZH2 overexpression, it was significantly increased (Fig. 4F; Fig. S1B). RT-qPCR results showed that EZH2 expression decreased following TUG1 knockdown but was rescued after EZH2 overexpression (Fig. S1C).

Next, we designed two shRNAs targeting ALYREF (shALYREF#1 and shALYREF#2) to evaluate whether ALYREF affected the expression of EZH2. RT-qPCR analysis showed that the expression of EZH2 was significantly reduced after ALYREF knockdown (Fig. 4G; Fig. S1D). EZH2 expression was increased in cells transfected with TUG1-overexpressing vectors but was decreased in those co-transfected with sh-ALYREF (Fig. S1F).

We further investigated the stability of EZH2 mRNA. Actinomycin D treatment significantly reduced the half-life of EZH2 after ALYREF depletion, which was reversed by TUG1 overexpression in SW1353 and JJ012 cell lines (Fig. 4H, I; Fig. S1E).

Because ALYREF is an important m5C reader, we explored the detailed mechanism underlying the m5C regulation of EZH2. First, we performed a methylated RIP assay to measure the m5C methylation level of EZH2 mRNA. As shown in Figure S2A, in CHS cells, the m5C methylation level of EZH2 mRNA was significantly higher than that in the negative control. Further RIP assays showed that DNA methyltransferase 3 beta (DNMT3B), which is thought to function in *de novo* methylation rather than maintenance methylation, bound to EZH2 mRNA in CHS cells (Fig. S2B). To verify the importance of EZH2 regulation by DNMT3B, we knocked down DNMT3B and assessed the expression of EZH2. The mRNA level of EZH2 was significantly decreased after DNMT3B knockdown (Fig. S2C, D). In addition, in the DNMT3B knockdown group, the m5C level of EZH2 was significantly reduced (Fig. S2E), and the enrichment of EZH2 and ALYREF was significantly reduced (Fig. S2F). Collectively, these findings indicate that DNMT3B is responsible for the m5C alteration of EZH2, while TUG1 enhances the stability of m5C-modified EZH2 RNA through the recruitment of ALYREF.

### lncRNA TUG1 represses CPEB1 expression via EZH2 in the nucleus

**Figure 5:**
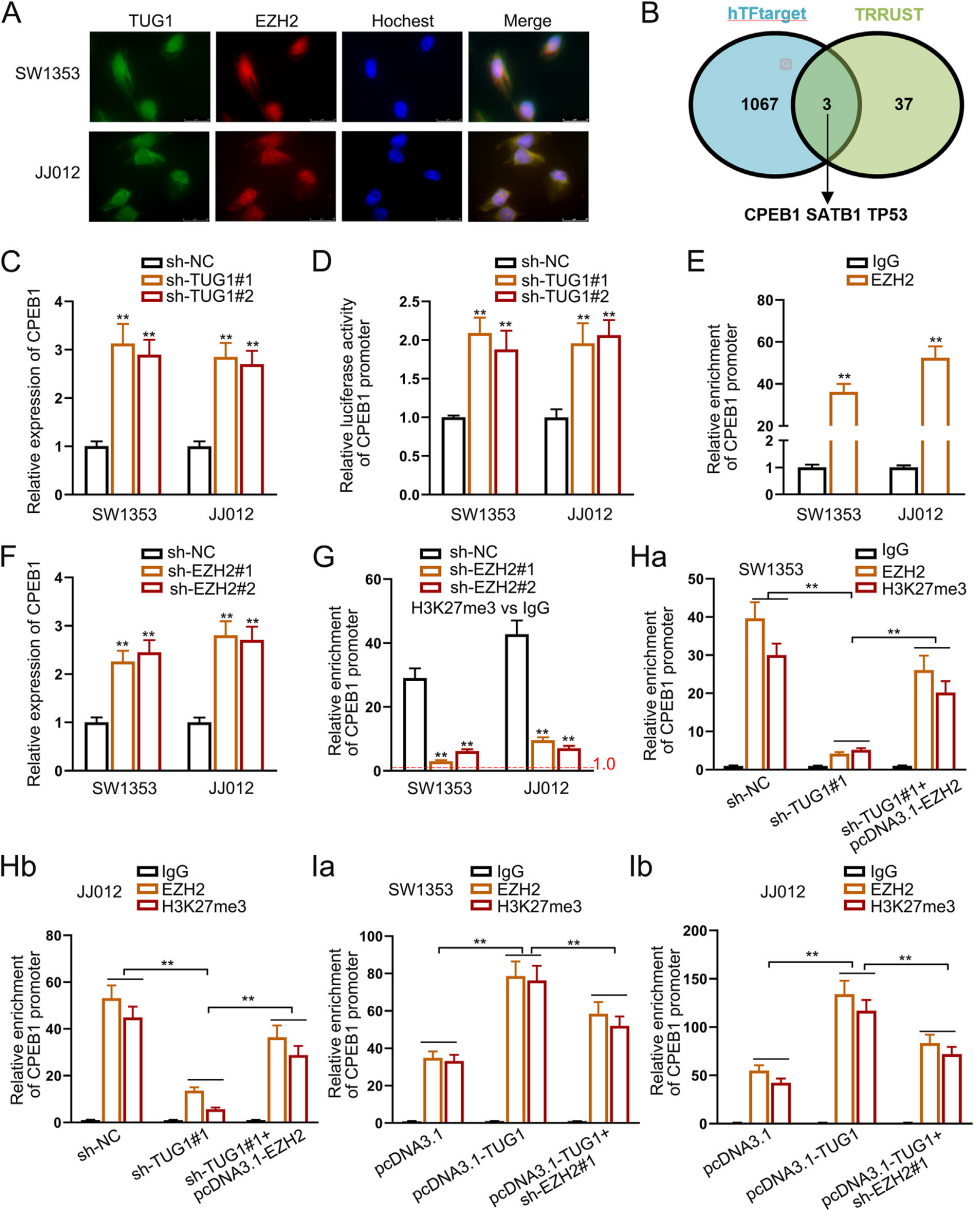
lncRNA TUG1 represses CPEB1 expression via EZH2 in the nucleus. **(A)** Immunofluorescence and fluorescence *in situ* hybridization assays showed the overlapping distribution of TUG1 and EZH2 in chondrosarcoma (CHS) cells. **(B)** The Venn diagram exhibiting the overlap of the target genes of EZH2 from hTFtarget and TRRUST. **(C)** Quantitative real-time PCR results of CPEB1 levels under TUG1 knockdown versus control in CHS cells. **(D)** Luciferase activity of CPEB1 promoter reporter in CHS cells with or without TUG1 knockdown. **(E)** Chromatin immunoprecipitation assay revealed that EZH2 bound to the CPEB1 promoter in CHS cells. **(F)** Quantitative real-time PCR results of CPEB1 levels under TUG1 knockdown versus control in CHS cells. **(G)** Chromatin immunoprecipitation assay revealed that knockdown of EZH2 led to decreased enrichment of CPEB1 promoter in H3K27me3 precipitates. **(H, I)** Chromatin immunoprecipitation assay revealed the enrichment of CPEB1 promoter in EZH2 or H3K27me3 precipitates under indicated transfection in CHS cells. The data is represented as mean ± standard deviation; *n* = 3; ∗*p* < 0.05, ∗∗*p* < 0.01.

To determine the downstream gene and signaling pathway regulated by EZH2 in CHS, we assessed the co-localization of TUG1 and EZH2 in SW1353 and JJ012 cells. TUG1 was co-localized with EZH2 in the nucleus of CHS cells (Fig. 5A). Based on the online databases hTFtarget and TRRUST, we identified three potential targets of EZH2, including CPEB1, STATB1, and TP53 (Fig. 5B; Supplementary File1). TUG1 knockdown only increased the expression of CPEB1 and had minimal effects on that of STATB1 and TP53, indicating that the regulation of CPEB1 by EZH2 is selective (Fig. 5C; Fig. S3A, B).

Considering the transcriptional regulation role of EZH2, we further identified the direct target of EZH2. The dual-luciferase reporter assay demonstrated that reducing TUG1 expression led to a notable rise in reporter activity compared with the control vector (Fig. 5D). Chromatin immunoprecipitation assays verified that EZH2 directly bound to the promoter region of CPEB1 (Fig. 5E).

Next, we evaluated whether EZH2 affected the expression of CPEB1. The RT-qPCR findings indicated that reducing EZH2 levels led to a notable increase in CPEB1 expression (Fig. 5F; Fig. S3C). Moreover, TUG1 knockdown suppressed EZH2 expression (Fig. S3D) and enhanced that of CPEB1, while EZH2 overexpression suppressed CPEB1 expression (Fig. S3E). Overexpression of TUG1 led to an increase in both the protein and mRNA levels of EZH2, as shown in Figure S3F and G, and reduced CPEB1 expression, while EZH2 knockdown rescued CPEB1 expression (Fig. S3H).

To verify whether EZH2 exerted its oncogenic role via H3K27me3 methylation, we conducted a chromatin immunoprecipitation assay using anti-IgG and anti-H3K27me3 antibodies in SW1353 and JJ012 cells with EZH2 depletion. H3K27me3 reduction in the promoter region of CPEB1 was observed in the EZH2 knockdown group (Fig. 5G). Silencing TUG1 led to a notable reduction in the binding of EZH2 to the CPEB1 promoter, which was reversed by the overexpression of EZH2. Moreover, the knockdown of TUG1 decreased the H3K27me3 level in the CPEB1 promoter but EZH2 overexpression increased the H3K27me3 level (Fig. 5H). However, TUG1 overexpression significantly increased the enrichment of EZH2 in the CPEB1 promoter, which was reduced by silencing EZH2. Moreover, TUG1 overexpression elevated the H3K27me3 level of the CPEB1 promoter, whereas EZH2 knockdown decreased the H3K27me3 level (Fig. 5I). These data indicated that TUG1 could repress CPEB1 expression through up-regulating the H3K27me3 level in the CPEB1 promoter region via EZH2, likely leading to CHS progression.

### Exosome-derived *TUG1* promotes M2 polarization

**Figure 6:**
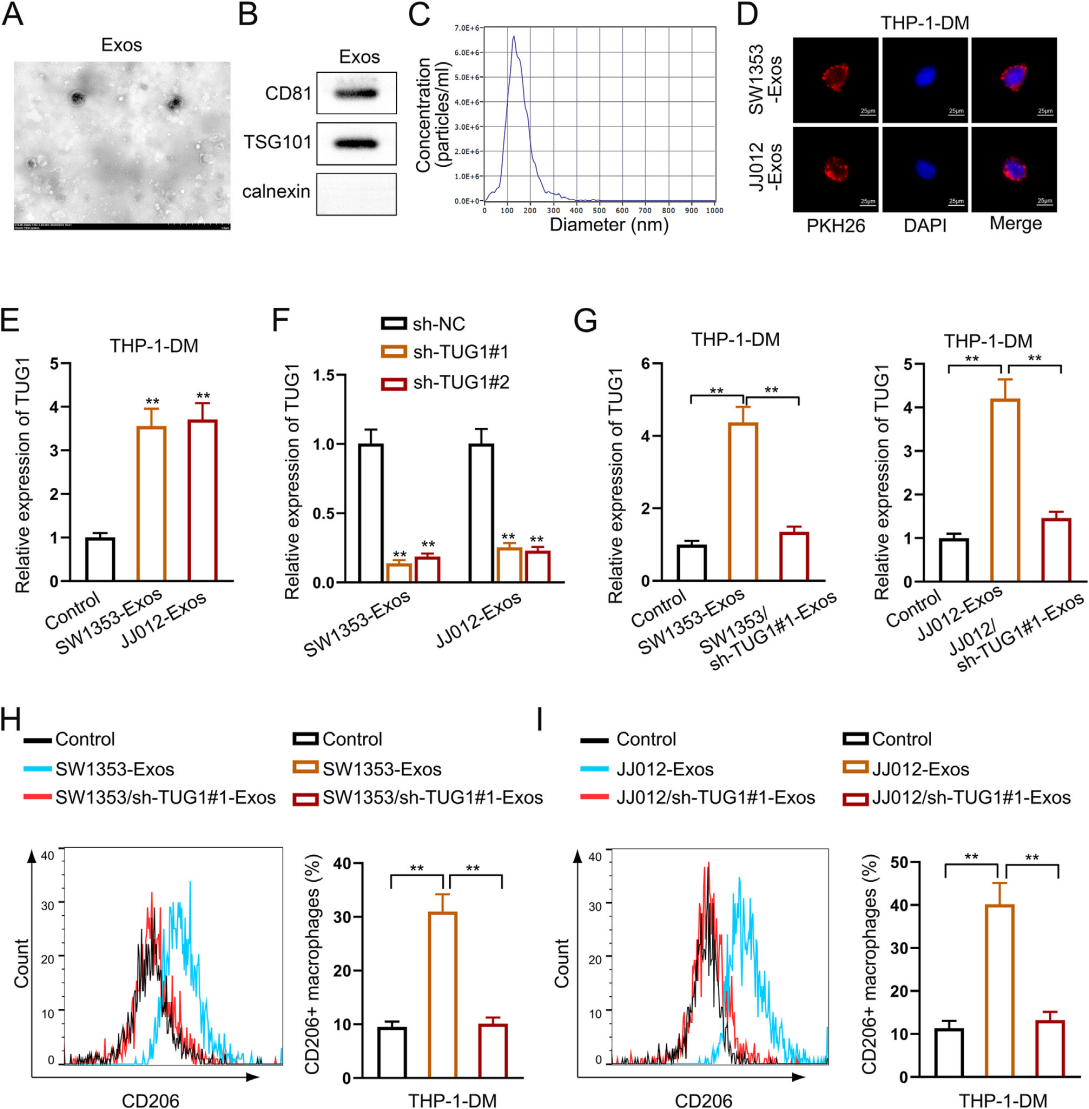
Exosome-derived TUG1 stimulates M2 polarization of macrophages. **(A)** Exosomes from the culture supernatant of chondrosarcoma (CHS) cells were observed via an electron microscope. **(B)** Exosome markers in CHS cells were observed by western blotting. **(C)** CHS cell exosome size was analyzed by nanoparticle tracking analysis. **(D)** Immunofluorescence was applied to detect the internalization of exosomes and THP-1. **(E)** The expression of TUG1 in THP-1 cells with or without CHS exosomes was measured by quantitative real-time PCR. **(F)** Quantitative real-time PCR was used to measure TUG1 expression in THP-1 cells by exosomes from CHS cells with or without TUG1 knockdown. **(G)** Quantitative real-time PCR analysis of TUG1 in THP-1 cells with or without TUG1 knockdown. **(H, I)** The expression of CD206 was analyzed by flow cytometry when CHS cells were co-cultured with THP-1 cells. The data is represented as mean ± standard deviation; *n = 3*; ∗*p* < 0.05, ∗∗*p* < 0.01.

Exosomes are crucial for cell communication as they transport lncRNAs and play a vital role in facilitating interactions between cells. Initially, exosomes were separated from the supernatants of CHS cells and characterized using transmission electron microscopy, western blotting, and nanoparticle tracking analysis. The average particles measured around 100 nm in size, with the presence of exosomal markers TSG101 and CD81 shown (Fig. 6A–C). These data indicate the successful isolation of CHS exosomes from the culture medium.

Next, we investigated the potential internalization of exosomes by macrophages. Confocal microscopy images showed that exosomes were taken up by THP-1-DM (Fig. 6D). The RT-qPCR analysis showed that TUG1 expression was significantly elevated upon treatment with CHS-derived exosomes (Fig. 6E). Meanwhile, the expression of TUG1 in THP-1-DM was dramatically decreased upon co-cultured with TUG1 knockdown CHS cells (Fig. 6F, G).

To investigate if exosomes from CHS could promote M2 polarization, CD206, a unique identifier for M2 macrophages, was utilized to differentiate the macrophage type in THP-1-DM cells through flow cytometry. Compared with untreated THP-1-DM, THP-1-DM treated with CHS-derived exosomes expressed significantly higher levels of CD206. Then, the exosomes extracted from the supernatants of CHS cells with TUG1 knockdown had lower CD206 levels (Fig. 6H, I). Lastly, GW4869 was used to inhibit the exosome secretion of CHS cells. CD206 expression was notably reduced in cells after GW4869 treatment (Fig. S4A, B). These data suggested that exosome-derived TUG1 stimulated M2 polarization when CHS cells co-cultured with macrophages.

### *TUG1* knockdown suppresses CHS metastasis and proliferation *in vivo*

**Figure 7:**
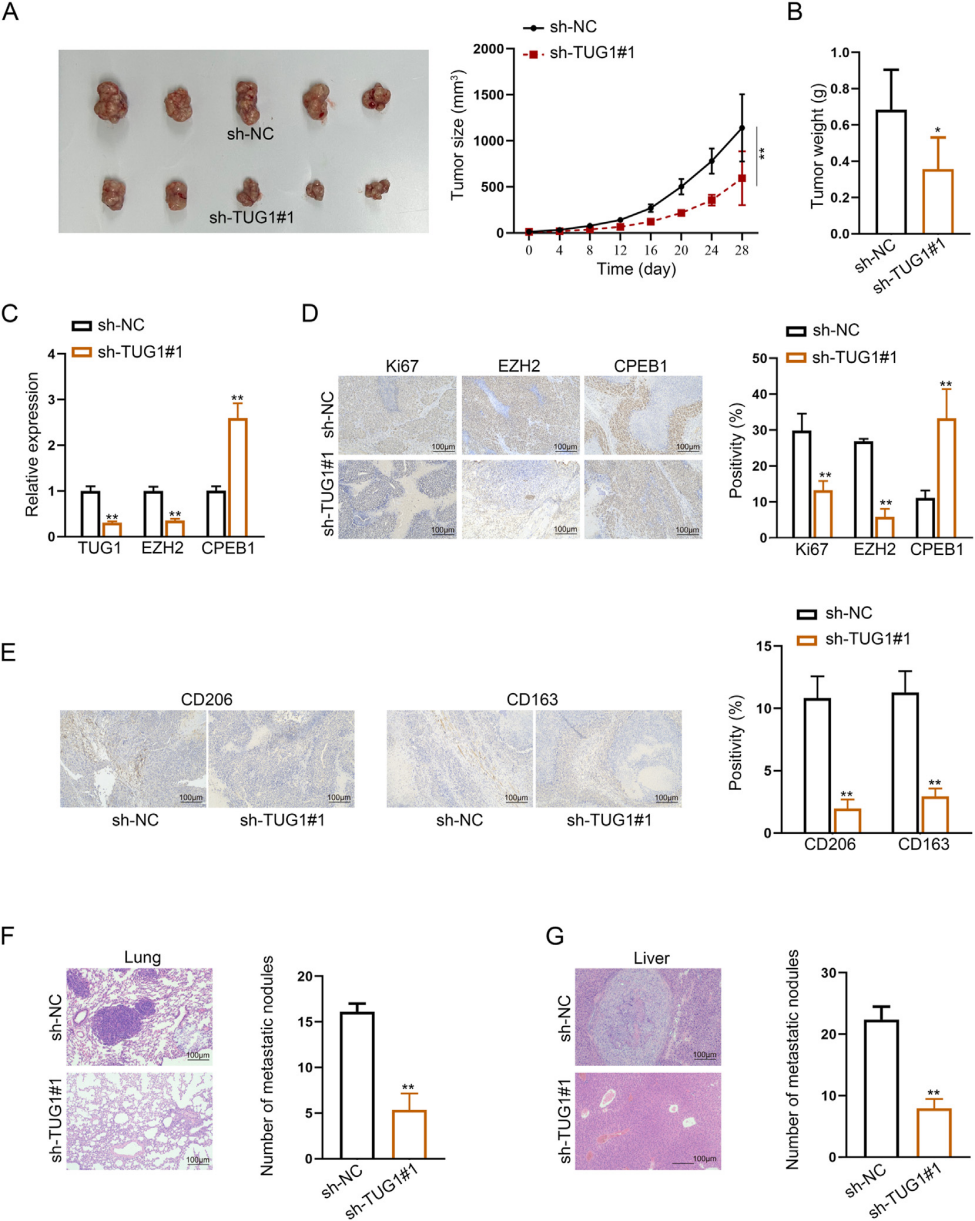
TUG1 knockdown suppressed chondrosarcoma (CHS) metastasis and proliferation *in vivo*. **(A)** Representative images of transplanted tumor tissues obtained from sacrificed mice 28 days after subcutaneous injection. Tumor size was measured with vernier calipers every 4 days. **(B)** The tumor weight obtained from sacrificed mice 28 days after subcutaneous injection. **(C)** Quantitative real-time PCR results of TUG1, EZH2, and CPEB1 levels under TUG1 knockdown versus control in xenografts derived from CHS. **(D)** Immunohistochemistry analysis of Ki-67, EZH2, and CPEB1 expression in xenografts derived from CHS under TUG1 knockdown versus control. **(E)** Immunohistochemistry analysis of CD206 and CD163 expression in xenografts derived from CHS under TUG1 knockdown versus control. **(F)** Lung invasion by SW1353 cells was evaluated by hematoxylin and eosin staining. **(G)** Liver invasion by SW1353 cells was evaluated by hematoxylin and eosin staining. The data is represented as mean ± standard deviation; *n* = 3; ∗*p* < 0.05, ∗∗*p* < 0.01.

To illustrate the function of TUG1 on CHS *in vivo*, we injected CHS cells with or without TUG1 knockdown subcutaneously into nude mice and monitored the tumor every four days. Silencing TUG1 reduced tumor size as well as tumor weight compared with the control group (Fig. 7A, B). Moreover, following TUG1 knockdown, EZH2 expression was dramatically decreased but CPEB1 expression was elevated in the excised tumor tissues (Fig. 7C). Immunohistochemistry staining revealed a reduction in Ki67 and EZH2 expression, along with an increase in CPEB1 expression, in the tumor tissues of mice injected with a cell mixture where TUG1 was knocked down (Fig. 7D).

The levels of M2 polarization markers CD206 and CD163 were decreased in the tumor tissue of mice injected with TUG1 knockdown cells (Fig. 7E). Hematoxylin and eosin staining of lung and liver tissues showed less infiltration and invasion after TUG1 knockdown (Fig. 7F, G). Collectively, the data indicated that silencing lncRNA TUG1 inhibited M2 macrophage polarization and attenuated the tumorigenesis of CHS *in vivo*.

## Discussion

**Figure 8:**
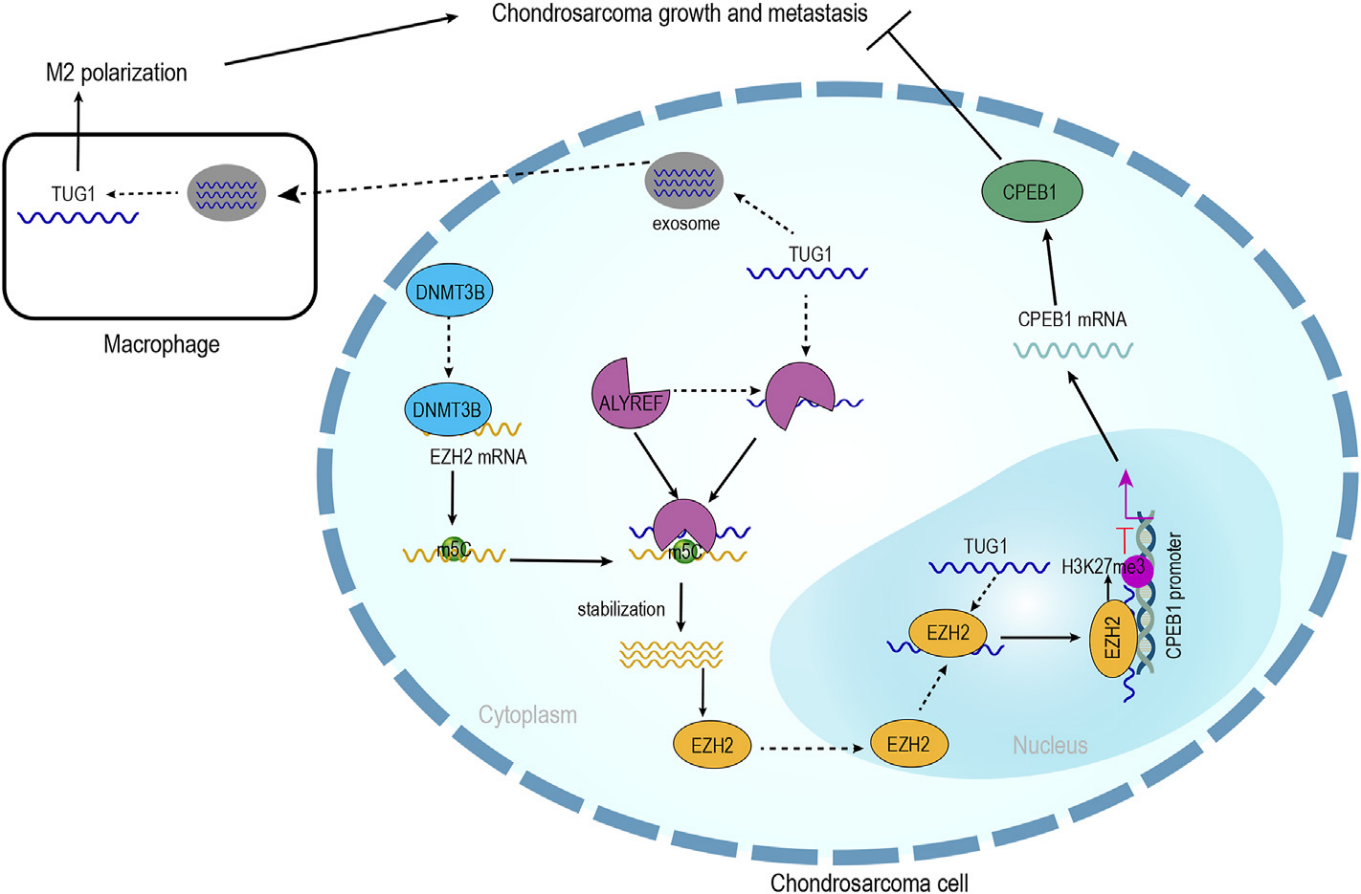
Mechanistic scheme of TUG1 in chondrosarcoma. TUG1 recruited ALYREF to maintain the stability of EZH2 by DNMT3B-mediated m5C modification. As a result, TUG1 facilitated the expression of EZH2, led to the repression of CPEB1 via H3K27me3 methylation, and ultimately promoted the progression of chondrosarcoma cells. Meanwhile, exosome-derived TUG1 transferred to tumor-associated macrophages and induced M2 polarization, thus, accelerating the progression of chondrosarcoma.

For the first time, this study revealed that TUG1 up-regulated EZH2 expression to suppress CPEB1 expression via H3K27me3 methylation, thereby promoting CHS progression. Additionally, exosome-induced TUG1 promoted the M2 macrophage polarization, leading to the advancement of CHS (Fig. 8). This study is believed to be the first one to elucidate the mechanism by which TUG1 functions in CHS, revealing its role in the progression of CHS.

TUG1 is crucial in the development and advancement of various human cancers by functioning as a competitive endogenous RNA that controls target genes, including miRNAs. As a miRNA sponge, TUG1 binds to several miRNAs. In renal cell carcinoma, inhibiting TUG1 reduced cell proliferation and movement by acting as a sponge for miR-299 to inhibit VEGF production.^19^ In hepatocellular carcinoma, TUG1 knockdown efficiently inhibited hepatocellular carcinoma cell proliferation and migration. Mechanistically, TUG1 acts as an endogenous “sponge” by binding to miR-328-3p, thus abolishing its role as a negative regulator of SRSF9 mRNA.^20^ In osteosarcoma, Xiao et al found that TUG1 silencing inhibited tumor growth, peritoneal spreading, and metastasis. Mechanistic studies revealed that TUG1 acted as a competitor to interact with miR-143-5p and counteracted the inhibition of HIF-1α expression caused by miR-143-5p.^21^ These findings indicated that TUG1 functioned as an oncogene through versatile pathways in distinct tumors. In osteosarcoma, Cao et al found that TUG1 regulated osteosarcoma development through miRNA-144-3p/EZH2/Wnt/β-catenin pathway.^22^ In bladder cancer, TUG1 also plays an important role. Researchers found that TUG1 regulated bladder cancer progression and metastasis by activating ANXA8 by sponging miR-140-3p, which provided a novel mechanism of bladder cancer pathogenesis.^23^ Lu et al reported that cancer-associated fibroblast-derived exosomal TUG1 promoted migration, invasion, and glycolysis in hepatocellular carcinoma cells via the miR-524-5p/SIX1 axis.^24^ Therefore, the regulation between TUG1 and other transcriptional factors in tumor development indicated that TUG1 might be deeply involved in the progression of CHS.

EZH2 functions as a component of the polycomb repressive complex 2, playing a crucial role in tumor progression and chemotherapy resistance by modifying H3K27me3 histones as a histone methyltransferase.^25^^,^^26^ Prior research has demonstrated that EZH2 executes its oncogenic function by transcriptional repression of tumor-suppressor genes. For example, in breast cancer, Li et al found that PRMT1-mediated meR342-EZH2 boosted the formation of PRC2 and inhibited the expression of PRC2 target genes p16 and p21 by blocking AMPK-mediated EZH2-T311 phosphorylation.^27^ Patil et al revealed that a lack of EZH2 in a transgenic mouse model of pancreatic ductal adenocarcinoma decreased the cancer’s occurrence and the development of liver metastases. Analysis of genome-wide occupancy and expression indicates that EZH2 plays a key role in suppressing transcriptional differentiation programs in pancreatic ductal adenocarcinoma, highlighting the importance of EZH2-mediated repression of GATA6 as a critical mechanism.^28^ In this study, we found that TUG1 recruited ALYREF to stabilize DNMT3B-mediated m5C modification of EHZ2. Subsequently, EZH2 repressed CPEB1 expression via H3K27me3 methylation, thus promoting the progression of CHS.

Increasing studies have shown that CPEB1 plays an important part in suppressing tumors. In hepatocellular carcinoma, Xu et al research has shown that increasing CPEB1 levels can decrease the growth of tumors, their ability to self-renew, and their resistance to chemotherapy in cases of hepatocellular carcinoma, as confirmed both *in vitro* and *in vivo* studies.^29^ miR-301 suppresses the expression of CPEB1 in breast cancer, which in turn regulates SOX2 expression through a SIRT1-dependent mechanism. Overall, miR-301 focuses on CPEB1 to boost the advancement of breast cancer through the SIRT1/SOX2 pathway.^30^ This study found that CPEB1 was down-regulated in CHS, which was suppressed by EZH2 H3K27me3 methylation.

Exosomes are essential for altering the tumor microenvironment and stimulating metastasis through the transfer of peptides or noncoding RNAs to nearby cells.^31^^,^^32^ lncRNAs found in exosomes can be internalized by nearby or faraway cells, influencing the behavior of the receiving cells. Extracellular lncRNAs enhance cancer development and advancement by controlling the interaction between cancer cells and macrophages. In pancreatic cancer, exosomal lncRNA FGD5-AS1 enhanced M2 polarization and interplayed with p300, a component of the STAT3/NF-κB signaling pathway, leading to heightened proliferation, migration, and invasion of pancreatic cancer cells.^33^ Colorectal cancer cells may receive lncRNA RPPH1 through exosomes, leading to the enhancement of macrophage M2 polarization and the stimulation of colorectal cancer cell growth. The process includes RPPH1 attaching to TUBB3, preventing its ubiquitination, boosting exosome-driven macrophage M2 polarization, and influencing the tumor microenvironment.^34^ Moreover, in renal cell carcinoma, Chen et al in their study demonstrated that exosomes from renal cell carcinoma could transport substantial amounts of lncRNA ARSR, leading to the transformation of macrophage phenotype from M1 to M2 and enhancing cytokine secretion, phagocytosis, and angiogenesis, thereby playing a crucial role in the progression of renal cell carcinoma.^35^ Our research showed that CHS-derived exosomal lncRNA TUG1 could drive the progression of CHS by inducing M2 polarization of macrophages. Further research is needed to investigate the precise roles of exosomal lncRNA-TUG1 and the molecular pathways through which TUG1 enhances CHS progression.

To sum up, this research showed that the expression of TUG1 was up-regulated in CHS, thus promoting the progression, migration, and invasion of tumor cells both *in vitro* and *in vivo*. Mechanistically, TUG1 recruited ALYREF to stabilize the DNMT3B-mediated m5C modification of EZH2, subsequently repressing CPEB1 expression via H3K27me3 methylation. This promoted the progression of CHS. Moreover, this study found that exosomes, crucial mediators of communication in the tumor microenvironment, were transported from CHS cells to macrophages to induce M2 polarization, hence accelerating CHS cell aggressiveness. However, the detailed mechanism of how tumor-associated macrophages are transformed into M2-type macrophages remains unclear; thus, this should be explored in subsequent research. To our knowledge, this is the initial research to reveal the complex regulatory network in CHS, offering possible treatment options for the disease in the future.

## CRediT authorship contribution statement

**Chao Li:** Writing – original draft, Methodology. **Wei Wang:** Software. **Binlong Zhong:** Validation, Supervision. **Lei Zhao:** Investigation, Formal analysis. **Juan Li:** Writing – review & editing. **Yihan Yu:** Methodology, Investigation. **Zhicai Zhang:** Supervision, Project administration. **Feifei Pu:** Writing – review & editing, Funding acquisition. **Jianxiang Liu:** Writing – review & editing, Supervision, Funding acquisition.

## Funding

This work was supported by grants from the 10.13039/501100001809National Natural Science Foundation of China (No. 82072978, 82274559, 82474545), the 10.13039/501100003819Natural Science Foundation of Hubei Province, China (No. 2024AFB1011), the 10.13039/501100002858China Postdoctoral Science Foundation (No. 2024T170247, 2024M750820), and the Natural Science Foundation of Wuhan, China (No. 2024040801020366).

## Conflict of interests

The authors declared no conflicting interests.
