# Supplementary material for: Long noncoding RNA TUG1 promotes chondrosarcoma progression and M2 polarization
Source: Genes Dis. 2024 Nov 30;12(4):101474. doi: 10.1016/j.gendis.2024.101474 (PMC12052688; doi:10.1016/j.gendis.2024.101474)
Supplement: Multimedia component 1 [file mmc1.docx]

The primers used in quantitative real-time PCR.

| Name | Sequence |
| --- | --- |
| TUG1 forward | CAACCATTTTGAAGCCCTGT |
| TUG1 reverse | GCTTTACACTGGGTGCCATT |
| U6 forward | TGATCTTCGGATCACTTTGG |
| U6 reverse | AATGGGTTTTCTCTCAATGTCG |
| GAPDH forward | CGACCACTTTGTCAAGCTCA |
| GAPDH reverse | AGGGGTCTACATGGCAACTG |
| EZH2 forward | AGGACGGCTCCTCTAACCAT |
| EZH2 reverse | CTTGGTGTTGCACTGTGCTT |
| DNMT3B forward | CACGCAACCAGAGAACAAGA |
| DNMT3B reverse | GGCAACATCTGAAGCCATTT |
| ALYREF forward | AAGTGGCAGCACGATCTTTT |
| ALYREF reverse | GTGCACGTCTGCTGTTCCTA |
| CPEB1 forward | TCTGCCCTTCCTGTCTCTGT |
| CPEB1 reverse | TATGCTGAAGGGGTCTTTGG |
| SATB1 forward | ACCAGTTCCAGGGAACACAG |
| SATB1 reverse | GGGGAGGGTGGTTCAAGTAT |
| TP53 forward | GGCCCACTTCACCGTACTAA |
| TP53 reverse | GTGGTTTCAAGGCCAGATGT |

shRNA sequences used in plasmid construction.

| shRNA name | Sequence |
| --- | --- |
| sh-TUG1#1 | TATATCATGGCAAGGAATGAACTCGAGCATTCCTTGCCATGATATATATTTTTG |
| sh-TUG1#2 | ATATAAACAAACTATTTCCAACTCGAGGGAAATAGTTTGTTTATATCCTTTTTG |
| sh-NC | CGGACCGUAGGACCAACCGUUCTCGAGAACGGTTGGTCCTACGGTCCGTTTTTG |
| sh-ALYREF#1 | GATCCGACGCCTATAATGCGAGAATGCTCGAGCATTCTCGCATTATAGGCGTCTTTTTG |
| sh-ALYREF#2 | GATCCGGAGTCTCAGACGCCGATATTCTCGAGAATATCGGCGTCTGAGACTCCTTTTTG |
| sh-DNMT3B#1 | GATCCAGCTGTCCGAACTCGAAATAACTCGAGTTATTTCGAGTTCGGACAGCTTTTTTG |
| sh-DNMT3B#2 | GATCCAGCCTAACACGGTGCTCATTTCTCGAGAAATGAGCACCGTGTTAGGCTTTTTTG |
| sh-EZH2#1 | GATCCCGGCTCCTCTAACCATGTTTACTCGAGTAAACATGGTTAGAGGAGCCGTTTTTG |
| sh-EZH2#2 | GATCCCGGCTCCTCTAACCATGTTTACTCGAGTAAACATGGTTAGAGGAGCCGTTTTTG |


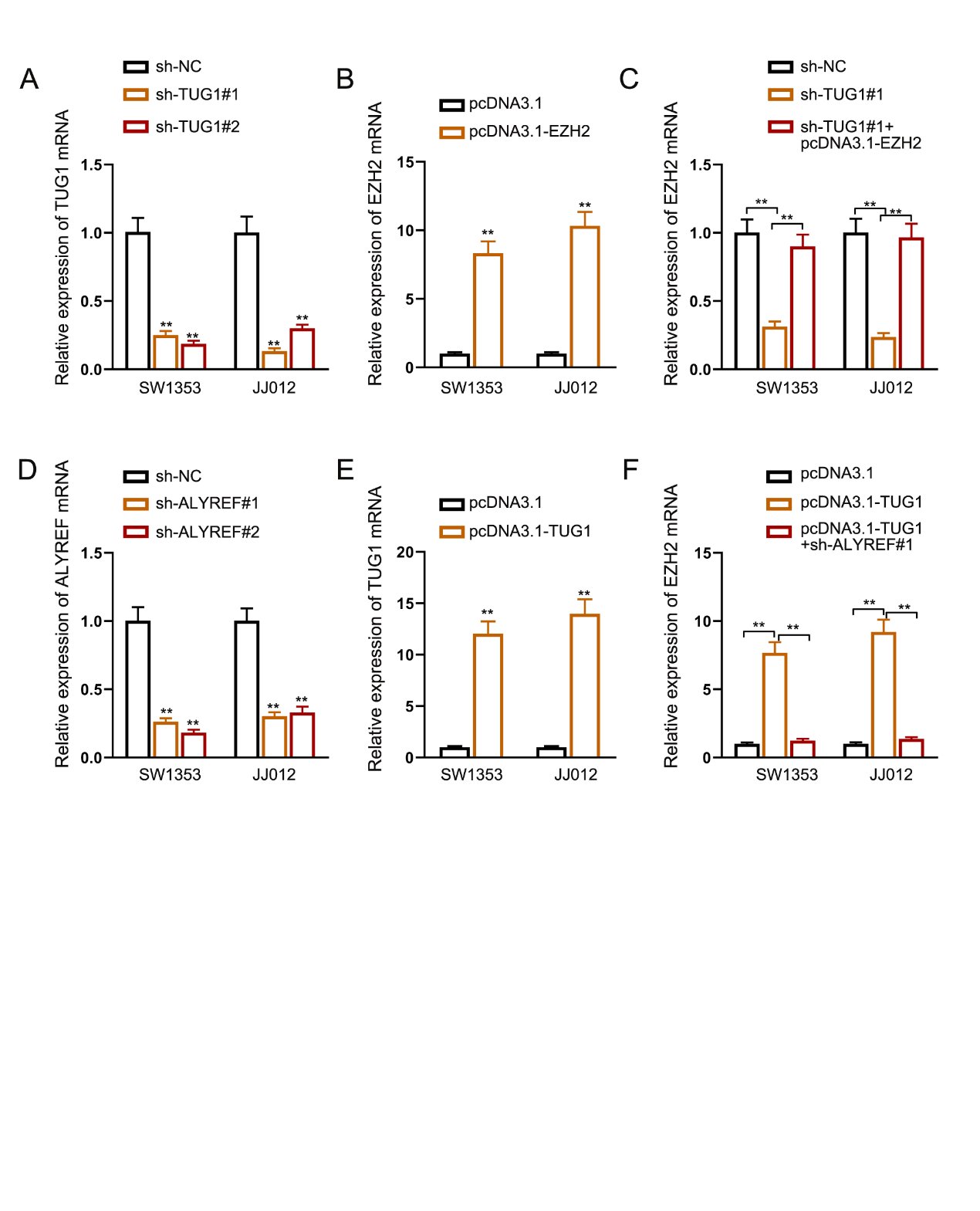


Figure S1

TUG1 regulated EZH2 expression in a ALYREF-dependent manner. (A) RT-qPCR analysis shows the knockdown efficiency of TUG1 in CHS cells. (B) RT-qPCR analysis shows the overexpression efficiency of EZH2 in CHS cells. (C) RT-qPCR confirmed the expression of EZH2 in TUG1-depleted CHS cells with or without EZH2 re-expression. (D) RT-qPCR analysis shows the knockdown efficiency of ALYREF in CHS cells. (E) RT-qPCR analysis shows the overexpression efficiency of TUG1 in CHS cells. (F) RT-qPCR confirmed the expression of EZH2 in TUG1-overexpression CHS cells with or without ALYREF knockdown. Data are shown as the mean ± SD of three independent experiments. (*p < 0.05; **p < 0.01;).


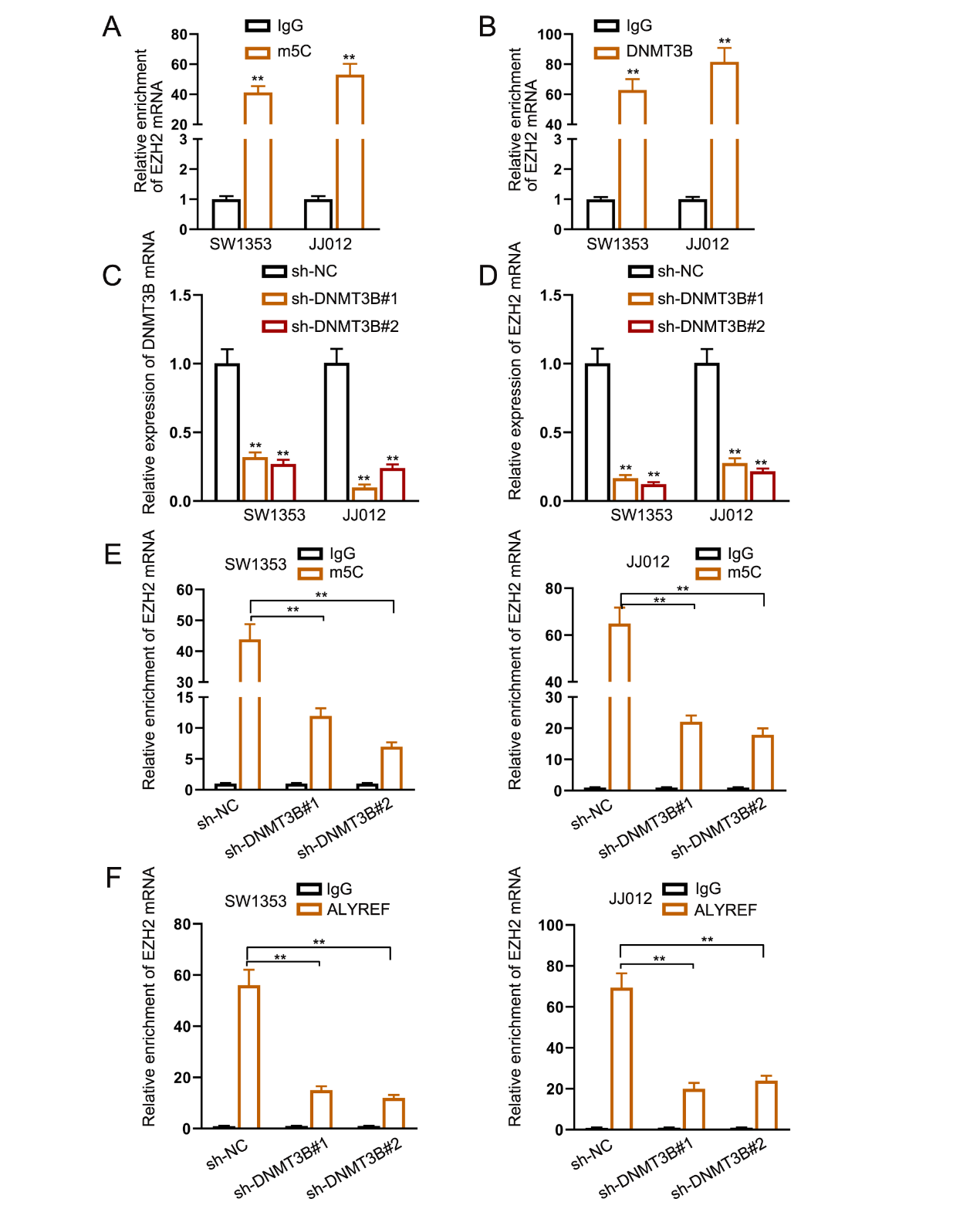


Figure S2

ALYREF was involved in DNMT3B-mediated RNA m5C modification of EZH2. (A) MeRIP assays detecting the RNA m5C modification of EZH2 in control and CHS cells. (B) RIP assay detecting the interactions between DNMT3B and EZH2 mRNA in SW1353 and JJ012 cells. (C) RT-qPCR analysis shows the knockdown efficiency of DNMT3B in CHS cells. (D) RT-qPCR confirmed the expression of EZH2 in DNMT3B-depleted CHS cells. (E) MeRIP assays detecting the RNA m5C modification of EZH2 in DNMT3B knockdown CHS cells. (F) RIP assay detecting the interactions between ALYREF and EZH2 mRNA in DNMT3B knockdown CHS cells. Data are shown as the mean ± SD of three independent experiments. (*p < 0.05; **p < 0.01;).


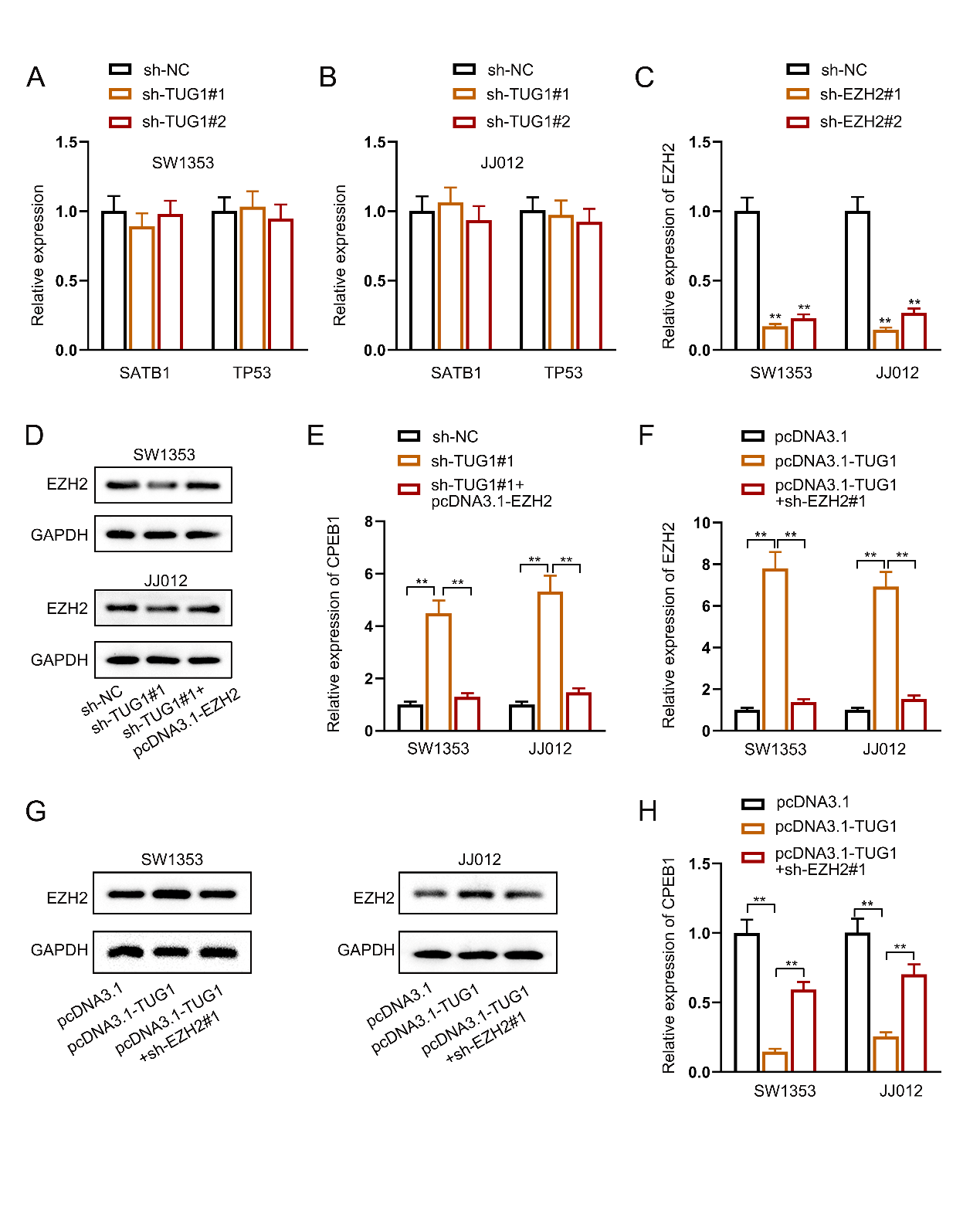


Figure S3

TUG1 regulated CPEB1 via EZH2. (A-B) RT-qPCR confirmed the expression of STAB1 and TP53 in TUG1-depleted CHS cells. (C) RT-qPCR analysis shows the knockdown efficiency of EZH2 in CHS cells. (D) Western blot confirmed the protein expression of EZH2 in TUG1-depleted CHS cells with or without EZH2 re-expression. (E) RT-qPCR confirmed the expression of CPEB1 in TUG1-depleted CHS cells with or without EZH2 re-expression. (F-G) qRT-PCR and Western blot confirmed the expression of EZH2 in TUG1-overexpression CHS cells with or without EZH2 knockdown. (H) RT-qPCR confirmed the expression of CPEB1 in TUG1-overexpression CHS cells with or without EZH2 knockdown. Data are shown as the mean ± SD of three independent experiments. (*p < 0.05; **p < 0.01;).


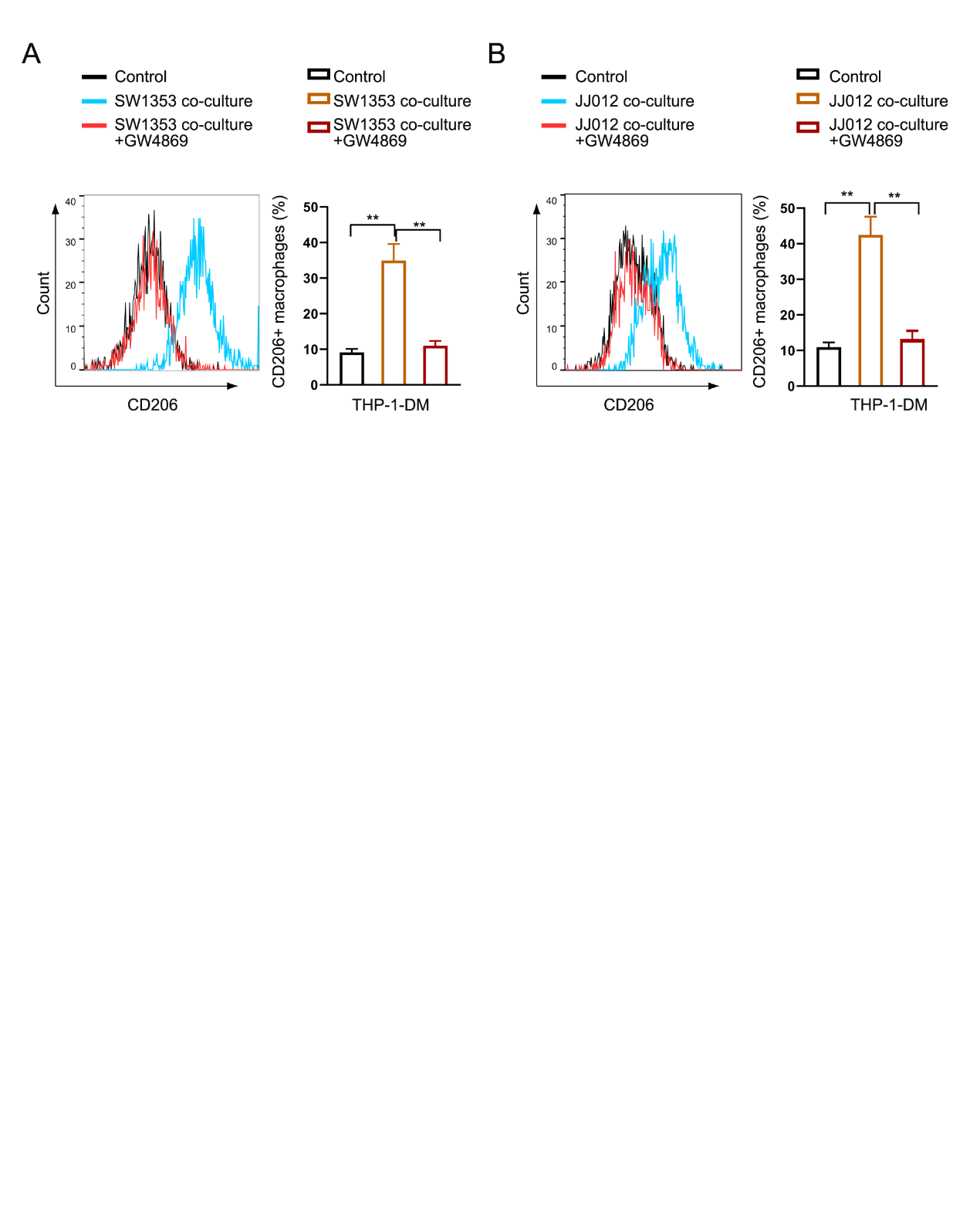


Figure S4

CHS cell-derived exosomal TUG1 induces M2 polarization of macrophages. (A-B) Flow cytometry analysis of CD206 in THP-1 cells co-cultured with CHS cells with or without GW4869 (an inhibitor of exosome secretion). Data are shown as the mean ± SD of three independent experiments. (*p < 0.05; **p < 0.01;).
